# Molecular insights into the *OGG1* gene, a cancer risk modifier in *BRCA1* and *BRCA2* mutations carriers

**DOI:** 10.18632/oncotarget.8272

**Published:** 2016-03-22

**Authors:** Carlos Benitez-Buelga, Tereza Vaclová, Sofia Ferreira, Miguel Urioste, Lucia Inglada-Perez, Nora Soberón, Maria A. Blasco, Ana Osorio, Javier Benitez

**Affiliations:** ^1^ Human Genetics Group, Spanish National Cancer Research Center (CNIO), Madrid 28029, Spain; ^2^ Familial Cancer Clinical Unit, Spanish National Cancer Research Center (CNIO), Madrid 28029, Spain; ^3^ Endocrine Cancer Group, Spanish National Cancer Research Center (CNIO), Madrid 28029, Spain; ^4^ Telomere and Telomerase Group, Spanish National Cancer Research Center (CNIO), Madrid 28029, Spain; ^5^ Spanish Network on Rare Diseases (CIBERER), Madrid 28029, Spain

**Keywords:** BRCA1 and BRCA2, telomere shortening, OGG1 polymorfism, cancer risk modifier, DNA damage

## Abstract

We have recently shown that rs2304277 variant in the *OGG1* glycosidase gene of the Base Excision Repair pathway can increase ovarian cancer risk in *BRCA1* mutation carriers. In the present study, we aimed to explore the role of this genetic variant on different genome instability hallmarks to explain its association with cancer risk.

We have evaluated the effect of this polymorphism on *OGG1* transcriptional regulation and its contribution to telomere shortening and DNA damage accumulation. For that, we have used a series of 89 *BRCA1* and *BRCA2* mutation carriers, 74 BRCAX cases, 60 non-carrier controls and 23 lymphoblastoid cell lines (LCL) derived from *BRCA1* mutation carriers and non-carriers.

We have identified that this SNP is associated to a significant *OGG1* transcriptional down regulation independently of the BRCA mutational status and that the variant may exert a synergistic effect together with *BRCA1* or *BRCA2* mutations on DNA damage and telomere shortening.

These results suggest that this variant, could be associated to a higher cancer risk in *BRCA1* mutation carriers, due to an *OGG1* transcriptional down regulation and its effect on genome instability.

## INTRODUCTION

Carrying an inherited mutation in the *BRCA1* or *BRCA2* genes increases a woman's lifetime risk of developing breast and ovarian cancers although there are considerable differences in disease manifestation. At the age of 70, cumulative cancer risk for *BRCA1* and *BRCA2* mutation carriers ranges from 43% to 88% for breast cancer development, and from 11% to 59% for ovarian cancer [1, 2].

In the context of *BRCA1* and *BRCA2* mutation carriers, it has been shown that other factors such as single nucleotide polymorphisms (SNPs) in genes from other DNA repair pathways could cause a higher genomic instability, hence increasing the cancer risk predisposition [3–6]. In this regard, a well-known synthetic lethal interaction is described between the *BRCA1* and *BRCA2* genes and the poly ADP ribose polymerase (*PARP1*), involved in the Base Excision Repair (BER) pathway [7]. BER corrects oxidative lesions in the DNA bases, which represent the major portion of endogenous DNA damage due to chemical reactions during cellular metabolism [8]. These lesions cause different types of DNA damage including DNA single-strand breaks (SSBs) or DNA double-strand breaks (DSBs) which are the principal source of genomic instability [9, 10]. In the presence of a defective *BRCA1* or *BRCA2* background, this accumulation of double-strand DNA breaks can persist and lead to cell cycle arrest or cell death; making BRCA-deficient cells extremely sensitive to PARP inhibitors (PARPi).

In addition, telomere instability/shortening occurring during oxidative and inflammatory stress can be explained by the strong tropism for guanine (G) oxidation at the telomere sequence (TTAGGG) [11]. For this reason, BER pathway is essential to maintain telomere integrity in mammals [12]. In fact, cellular changes due to BER defects have been implicated in a multitude of diseases, ranging from cardiovascular diseases, arthritis, cancer, as well as aging and age-related disorders [13, 14].

SNPs in genes involved in the BER pathway have been reported to modify ovarian and breast cancer risk in *BRCA1* and *BRCA2* mutation carriers. In particular, one of the most recent examples was described by our group for a SNP (rs2304277) in the *OGG1* (8-guanine DNA glycosylase) gene that was associated with increased ovarian cancer risk in *BRCA1* mutation carriers [5]. The *OGG1* gene encodes for a key enzyme involved in the first steps of BER that removes a highly mutagenic base, 8-oxodeoxyguanosine, generated by oxidative stress [15].

In this study, by using two independent sample sets with different *BRCA* status, we have explored the role of this polymorphism on *OGG1* transcriptional regulation and its possible implication on genome instability. With this, we would like to explain the cancer risk modifier effect that this gene exerts in carriers of *BRCA1* and *BRCA2* mutations.

## RESULTS

### SNP frequency in FBOC and LCL

We genotyped the SNP rs2304277 in both, FBOC and LCLs sample sets, to perform genotype/phenotype studies (role of the SNP on: *OGG1* mRNA expression, telomere studies, and DNA damage). In the FBOC samples, we identified 36% of the samples (81/223) carrying the variant. The same frequency was reported in our previous study analyzing more than 23000 cases and controls [5].

The different group of cases and controls presented similar frequencies that are summarized in Supplementary Table S1. No significant differences were found among groups.

From a total of 23 cell lines, 9 harbored the SNP (39%). From 16 of the LCL with *BRCA1* mutation 7 LCL harbored the SNP (43%) and from the 7 non-carrier controls, 2 had the variant (33%) (Supplementary Table S2).

### Expression of *OGG1* in FBOC, Gtex server and LCLs

**Figure 1:**
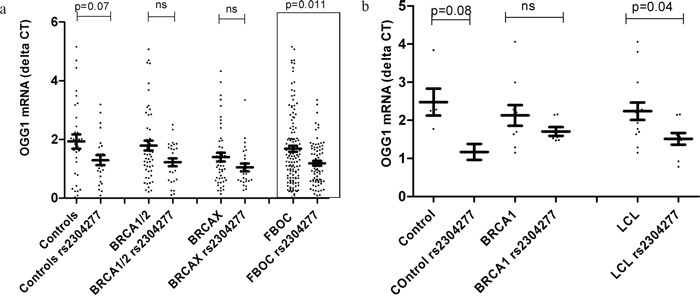
**a. Comparative analysis relative to the *OGG1* mRNA expression levels between FBOC groups (*BRCA1 & BRCA2,* BRCAX) and controls according the presence of the *OGG1* SNP.** Control group harbouring the variant showed a statistical trend of lower *OGG1* mRNA levels (p=0.07); while we didn't detect significant differences in *OGG1* transcriptional levels within *BRCA1* and *BRCA2* group due to the presence of the SNP (p=0.11). When all FBOC samples, were stratified according to the presence of the SNP, we observed significant lower *OGG1* mRNA expression levels in the individuals harbouring the variant (p=0.011). Line at mean with standard error mean (SEM) **b. Transcriptional mRNA basal levels of *OGG1* in Lymphoblastoid cell lines (LCLs)**. Each dot at the graph, represent the mean OGG1 mRNA values from two independent measurements (two clones of each sample) for most LCL analyzed (20/23), for 3 samples we could measure only once. We found that LCL harbouring the SNP presented significant lower *OGG1* mRNA levels when compared to those who did not harbour the SNP (p=0.04). Line at mean with standard error mean (SEM).

In order to know if the SNP could affect gene expression, we first analyzed in the FBOC series the *OGG1* mRNA expression levels considering both, the BRCA mutational status and the presence or absence of the *OGG1* variant to stratify and compare expression values among groups (Figure 1a).

First, we did an independent lineal regression analysis in BRCA1/2 mutation carriers to test whether cancer status (individuals with or without cancer antecedents) could affect *OGG1* mRNA levels; because it did not affect, we decided to include healthy and affected BRCA1/2 mutation carriers in the same group (BRCA1/2) for expression studies, Supplementary Table S3.

In the comparative analysis, we detected an *OGG1* mRNA down regulation in individuals harboring the variant. This down regulation was statistically significant when we stratified all the FBOC individuals by the presence of the variant (with/without) regardless the BRCA status (BRCA1/2, BRCAX and non-carrier controls), p=0.011. Although. we were not able to detect significant differences within each mutational group (non carrier controls, *BRCA1/2* and BRCAX*)* probably due to the reduced sample size (Figure 1a); a complementary lineal regression analysis confirmed a significant down regulation associated to the SNP in the non carrier controls (β=−0.63);p=0.049), in BRCA1/2 (β=−0.57;p=0.027) and a trend in the BRCAX group(β=−0.34;p=0.123), suggesting that the variant could be associated *per se* to lower *OGG1* mRNA levels independently of the BRCA mutational group.

In parallel, we tested *in silico* the SNP effect on transcriptional regulation in different tissues using the Gtex eQTL web server (http://www.gtexportal.org). Interestingly, we observed down regulation for whole blood, uterus, vagina and ovary, but only the last one presented a significant *OGG1* transcriptional down regulation (p=0.023), Supplementary Table S4. Ovary is the tissue where this variant was originally found to be associated to an increased cancer risk [5].

Finally, we measured *OGG1* mRNA basal levels among the 23 LCL considering the BRCA status, and presence or absence of the SNP. Only when we group all LCL together considering the presence of the SNP we are able to detect significant down regulation (p=0.04) Figure 1b, probably because the sample size was too small to detect significant association p-values of *OGG1* mRNA down regulation within groups (*BRCA1* non carriers LCLs and *BRCA1* LCLs).

### Telomere length studies in FBOC

We explored the role of this variant on TL maintenance. Hence we measured TL and percentage of short telomeres by HT QFISH in the blood cells from FBOC patients and non-carrier controls to establish genotype/phenotype associations.

We first evaluated the TL distribution in 60 healthy women as a function of age to obtain a regression line to adjust the TL from FBOC samples. As expected, we found a decrease in TL with age, Supplementary Figure S1.

Because mean TL is strongly heritable [16] and our series contains related individuals, we used a single member (genotype) from each family for both, BRCA status and presence or absence of the SNP for the analysis. Whenever possible, we used the index-case of the family and if this sample was not available, we used the latest genotype included in the family as common criteria of the study.

Chemotherapy status, another possible confounding factor that alters TL was corrected to perform this analysis [17]. We eliminated those cancer patients who were undertaking chemotherapy or those within a window of 2 years since the last cycle of chemotherapy. In total, 13 BRCA1/2 cases and 26 BRCAX cases were excluded.

Hence, we used a total of 44 controls (19 harboring the SNP), 21 *BRCA1* carriers (10 harboring the SNP), 28 *BRCA2* carriers (9 harboring the SNP), 1 patient harboring mutation in both genes and 38 BRCAX (15 harboring the SNP).

First, we did an independent lineal regression analysis in BRCA1/2 mutation carriers to test whether cancer status (individuals with or without cancer antecedents) could affect TL and percentage of short telomeres; because it did not affect these 2 factors (Supplementary Table S3), we decided to include healthy and affected BRCA1/2 mutation carriers in the same group (BRCA1/2) for telomere studies.

**Figure 2:**
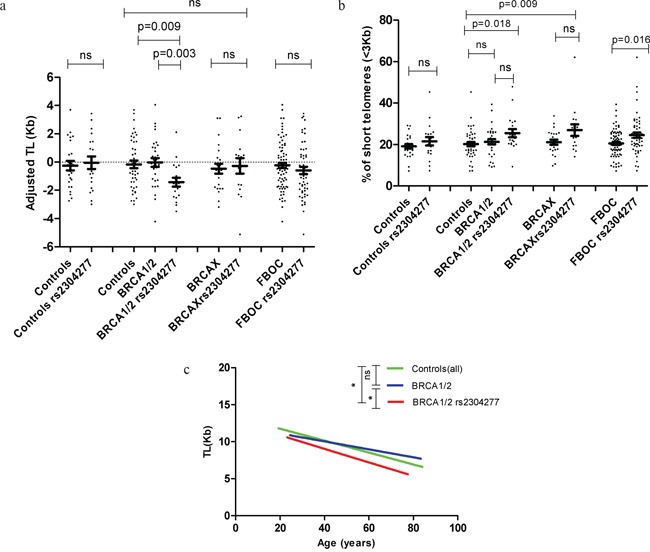
**a. Distribution of the TL (Kb) values adjusted for age according to mutational status.** We did not detect significant differences in TL for the control group due to the presence of the SNP; while TL was significantly shorter in BRCA1/2 mutation carriers harbouring the variant when compared to non carriers of the SNP (p=0.003) or controls(0.009). We were not able to find any difference in TL among the BRCAX group of patients. Additionally we stratify all the FBOC samples according to the presence of the variant and we did not detect any significant difference in TL between carriers and non carriers. Line at mean with standard error mean (SEM) **b. Comparative analysis among FBOC genotypes regarding the percentage of short telomeres (<3Kb)**. We did not detect significant differences in the percentage of short telomeres neither in the control nor in the BRCA1/2 groups when the variant was present; however BRCA1/2 or BRCAX harbouring the variant presented significant higher % of short telomeres compared to controls(all)(p=0.018; p=0.009). Additionally we stratify all the FBOC samples according to the presence of the variant, and we could detect a significant higher % of short telomeres in those samples harbouring the SNP (p=0.016) Line at mean with standard error mean (SEM) **c. Telomere shortening lines in BRCA1 or BRCA2 mutations carriers group (BRCA1/2) with and without the variant, and the non-carriers controls.** TL (Kb) is represented in this graph according to age (years). Regression line is draw in green colour for controls (y=−0.080*age+13.367), blue colour for BRCA1/2 patients (y=−0.537*age+12.188) and red colour for BRCA1/2 with the variant (y=−0.0918*age+12.705). F-test: BRCA1/2 *vs* BRCA1/2 rs2304277 (p=0.010); Controls *vs* BRCA1/2 rs2304277 (p=0.034).

In the comparative analysis, Mann Whitney U test revealed no significant differences neither in adjusted TL nor in percentage of short telomeres between controls harboring and not harboring the variant (Figure 2a & Figure 2b). However, we observed significant shorter TL among BRCA1/2 carriers harboring the variant compared to those BRCA1/2 carriers not harboring the SNP (p=0.003) or controls (p=0.009), Figure 2a. Additionally, increased percentage of short telomeres were detected in BRCA1/2 mutation carriers harboring the SNP compared to the control group (p=0.018), Figure 2b. In the group of BRCAX cases we did not detect any effect of the SNP on TL although we found a significant increased percentage of short telomeres (p=0,009) compared to controls, Figure 2a and Figure 2b.

Then, we analyzed all FBOC patients together considering the presence/absence of the variant to test the effect of the SNP alone, regardless the BRCA mutational status. We were not able to observe significant differences on TL but we detected a significant increased percentage of short telomeres in the group harboring the SNP (p=0.016), Figure 2a and Figure 2b.

Linear regression analysis revealed that TL was significantly modified by the presence of the SNP in the group of patients harboring mutations in *BRCA1* or *BRCA2* genes (BRCA1/2) (β= −1.438; C.I (−2.554 – (−0.323); p=0.013), Supplementary Table S3; but not in the non-carrier controls or BRCAX groups (data not shown).

Hence, we stratified BRCA1/2 patients according to the SNP and we compared the linear model between each of the groups (BRCA1/2 with/without the SNP and controls). Significant differences were detected in BRCA1/2 carriers harboring the SNP when compared to those not harboring the SNP (p=0.010) or controls (p=0.034), Figure 2c. In fact, we observed a faster telomere shortening (slope) in the group of patients harboring both, the BRCA1/2 mutation together with the SNP, compared to those who did not harbor the SNP, or the control group, Figure 2c (legend).

### Telomere length study in LCLs

We compared telomere shortening during normal replication among the *BRCA1* LCLs to confirm experimentally the faster telomere shortening (slopes) observed in the FBOC patients who harbored the BRCA1/2 mutation together with the variant. Additionally, we measured and compared the accumulation of short telomeres along the cell culture.

Due to technical issues and the differences in growth rate, we could only use a set of 8 out of the 16 LCLs harboring mutation in *BRCA1* gene to follow the telomere shortening during 55 passages. From the 8 LCL with mutation in *BRCA1* gene 3 presented also the SNP.

**Figure 3:**
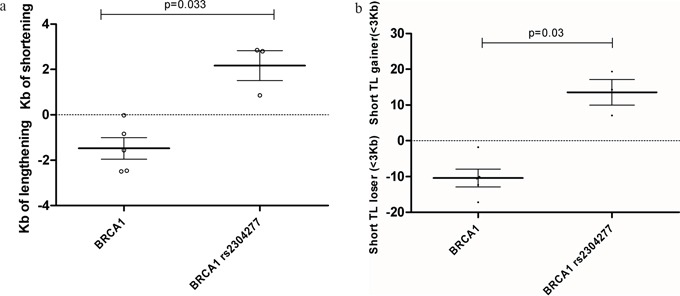
We measured TL differences between passage n°1 and passage n°55 for each LCL, to calculate telomere shortening/ lengthening in Kb and the gain or lose of critical short telomeres(<3Kb) **a. Telomere length lose or gain after 55 passages of culture among *BRCA1* mutated LCLs.** Significant telomere shortening in LCL harbouring *BRCA1* mutation together with the SNP was detected after 55 passages compared to those not harbouring the variant (p=0.033). Line at mean with standard error means (SEM) **b. Percentage of critical short telomeres gain or lose after 55 passages of culture among among *BRCA1* mutated LCLs.** Significant increased amount of short telomeres was found in the LCL harbouring BRCA1 mutation together with the SNP after 55 passages compared to those not harbouring the variant (p=0.03). Line at mean with standard error means (SEM).

Our results revealed significant faster telomere shortening after 55 cell culture passages, in the group of samples harboring *BRCA1* mutation together with the SNP (p=0.033). This result is similar from the previous obtained in patients suggesting that this event is taking place only when *BRCA1* mutation and the variant are together, Figure 3a. We could also confirm a significant accumulation of short telomeres in the LCL harboring the *BRCA1* mutation together with the variant after 55 passages of cell culture (p=0.03), Figure 3b.

### DNA damage

To test the possible contribution of the SNP to a higher DNA damage we measured the mean γH2AX intensity signal in the cell nucleus at basal conditions (first passage and no irradiation).

**Figure 4:**
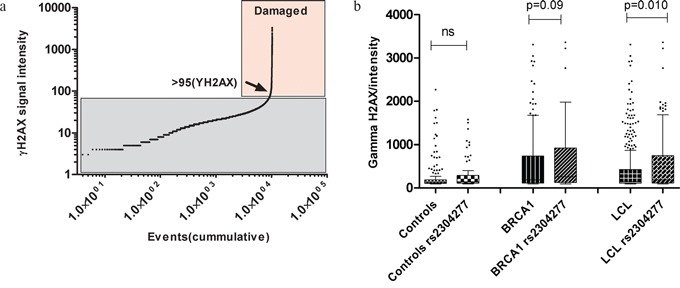
**a. Threshold of γH2AX nuclear intensity damage.** We selected the intensity value of 95(arbitrary units) as a cut of to establish the damaging signal intensity because this was the value in where the distribution change shape exponentially, indicating which are the normal and the abnormal (damaging)values. **b. Comparative analysis regarding the signal intensity of γH2AX at the nucleus among the LCL genotypes.** The effect of the variant is not significant in the control group (p>0.05) while in the group of cells carrying mutation in BRCA1, we found higher γH2AX signal intensity when the variant was present (p = 0.09). We stratified all the 23 LCL according the presence of the variant and we detected significant higher γH2AX intensity in the carriers of the variant (p =0.010). Line at mean with standard error means (SEM).

We plotted all the γH2AX values from the LCL in a cumulative frequency histogram to establish a damage threshold above which we observed an exponentially increase in the γH2AX intensity values, which indicates the cells with a clear nuclear DNA damage. We established the threshold in 95 arbitrary units of γH2AX of nuclear intensity (Figure 4a).

Then, we calculated the frequency of damaged cells among LCLs with different genotypes and the intensity of the nuclear γH2AX signal in these cells to evaluate the possible impact of the *OGG1* SNP on DNA damage. We found minimum differences in the percentage of damaged cells associated to the presence of the SNP (5.8% and 6.3% in LCLs with and without the SNP, respectively). However, the intensity of the damage was significantly higher in LCLs harboring the SNP (p=0.010) compared to those not harboring the variant, Figure 4b.

## DISCUSSION

We have previously found that the *OGG1* SNP rs2304277may be a modifier of cancer risk in *BRCA1* mutation carriers [5]. *OGG1* belongs to the BER pathway that plays an important role correcting DNA lesions originated by oxidative stress. These lesions are the principal source of genomic instability and can drive to cancer development. In this study we have shown how this variant can contribute to increase cancer risk in *BRCA1* carriers, by reducing the mRNA *OGG1* expression levels, increasing the DNA damage as a consequence of genomic instability generated, and shortening the telomeres in a synergic way with the *BRCA1* mutation.

Because rs2304277 is located 1.8Kb downstream of 3′UTR region of the *OGG1* and post transcriptional modifications, like potential illegitimate microRNA target site [18, 19], could be altering normal *OGG1* mRNA regulation, we decided to explore the role of this SNP on transcriptional regulation using two set of samples. The first set consisted in 223 blood samples from controls and FBOC patients with a heterogeneous BRCA mutational status (*BRCA1, BRCA2* and BRCAX) and the second was a panel of 23 LCLs derived from *BRCA1* mutation carriers and non-carrier controls. The percentage of heterozygotes for the SNP in the FBOC and LCL set of samples was 36% and 39%, respectively, which was the expected frequency [5].

We confirmed in both sample sets (FBOC series and LCL) significant lower expression of *OGG1* mRNA transcript associated to the SNP, independently of BRCA mutational status, (Figure 1a & Figure 1b). We extended the analysis using Gtex eQTL dB (http://www.gtexportal.org) looking for the SNP effect over *OGG1* mRNA levels in different tissues and we found significant down regulation in ovary (p=0.023) tissue where this SNP was initially found to be associated to a higher cancer risk, Supplementary Table S4.

These results suggest that this cancer risk variant is likely associated with mRNA *OGG1* transcriptional down regulation which can potentially lead to higher genome instability due to a defective 8-oxoG repair capacity. In this way, the aberrant accumulation of 8-oxoG was previously associated with faster development of lung adenocarcinoma in *OGG1* knock-out mice models [20] while in transgenic mice it was demonstrated that over expression of *OGG1* attenuated breast cancer progression and metastasis through a reduction in the oxidative damage [21]. All these data suggest a critical role of this gene in cancer development and progression which, together with BRCA mutations could result in higher genome instability and increased cancer risk.

Given the role of the BER pathway and in particular the *OGG1* enzyme on telomere repair [11, 22], we explored the impact of this SNP on some features related to telomere biology considered as hallmarks of genome instability, such as telomere shortening or the percentage of critically short telomeres. We found in the linear regression analysis, that the SNP may be a TL modifier for *BRCA1* and *BRCA2* mutations carriers (p=0.013). Carriers of BRCA1/2 mutations and *OGG1* SNP presented a significant shorter TL compared to controls (p=0.009) and mutation carriers not harboring the SNP (p=0.003) (Figure 2a), likely due to an accelerated telomere shortening during life-time (Figure 2c). We also found an increase of short telomeres in those individuals harboring the SNP, regardless the BRCA mutational status (p=0.016) (Figure 2b).

These results were experimentally validated in our LCL set by measuring TL after 55 passages. We could confirm a significant faster telomere shortening in the group of samples harboring a *BRCA1* mutation together the SNP (p=0.033) (Figure 3a), which correlated with a significant accumulation of short telomeres after a total of 55 cell culture passages (p=0.03) (Figure 3b). Our results point to a synergistic effect of the SNP and the *BRCA1* mutation on telomere shortening. This telomere instability may be due to the cell tropism for the accumulation of oxidative lesions at the telomeric region [23, 24] in the context of defective BER performance [22] triggered by the SNP effect on *OGG1* down regulation. In this sense other authors have reported that SNPs located in the 3′UTR region of *OGG1* could be associated with a lower 8-oxoG repair activity being particularly sensitive to the cellular redox status. [25, 26]

The region represented by the SNP has been previously spotted by other authors who also found associations with different cancer types [27–29]. Then, we tested whether this SNP could have an impact on DNA damage, measured in this case by γH2AX, a DNA damage marker of DSB [30].

Using the LCL panel, we compared the percentage of damaged cells and its nuclear γH2AX signal intensity among different genotypes at basal conditions (first passage and no irradiation). Despite we found a similar percentage of damaged cells among LCLs with and without the variant (5.8% and 6.3%, respectively), we could detect that those LCLs harboring the SNP, presented significantly higher γH2AX signal intensity at the nucleus, pointing to a more profound DNA damage (p=0.010) (Figure 4b). These results are similar to other reported in the literature establishing association between SNPs in *OGG1* at the same gene region with an increased DNA damage/genome instability due to an impaired BER performance [6, 25-27, 31, 32]

In summary, we have identified that the *OGG1* SNP itself contributes to a higher nuclear DNA damage intensity, probably due to a defective BER performance triggered by *OGG1* transcriptional down regulation. Additionally, our results suggest a synergistic effect between *BRCA1* or *BRCA2* mutations with the SNP rs2304277 on specific telomere instability hallmarks, such as telomere shortening and the accumulation of short telomeres, when both genetic events are present in the cell. These molecular processes could explain the relation between this SNP and *BRCA1* or *BRCA2* mutations, on cancer risk.

## MATERIALS AND METHODS

### Familial breast and ovarian cancer (FBOC)

We studied two different set of samples: A first group, was composed by 223 individuals belonging to 121 families meeting high risk criteria and screened for deleterious mutations in *BRCA1* and *BRCA2* genes, as previously reported [1]. 24 carried a deleterious mutation in *BRCA1*, 25 in *BRCA2*, 1 family harbored both *BRCA1* and *BRCA2* mutations and 71 did not carry any mutation (*BRCAX*).

Sixty individuals were used as non-carrier controls: They were relatives of BRCA1/2 carriers, who didn't have any personal cancer antecedent and didn't harbor the corresponding familial mutation in *BRCA1* or *BRCA2* genes. General characteristic of this series are described in Table 1.

**Table 1:** Description of the analyzed series and the different studies performed

Families (n)	Healthy carriers	Affected carriers	^c^ Non-carriers controls	Total	Median age, (range)	SNP Genotyping	Expression studies	^d^TL studies
BRCA1, (24)	18	20	13	51	45, (23-78y)	51	48	30
BRCA2, (25)	27	21	25	73	50, (22-87y)	73	64	46
^a^BRCA1 + BRCA2, (1)	1	2	1	4	54, (42-61y)	4	4	3
^b^ BRCAX, (71)	-	74	21	95	49, (20-85y)	95	92	53
Total FBOC, (121)	46	117	60	223	49, (18-87y)	223	209	132

a Refers to a family harboring mutations in both BRCA1 and BRCA2 genes.

b Non BRCA1 or BRCA2 families.

c Non carrier controls were composed by family relatives without any antecedents of cancer and negative for BRCA1 or BRCA2 mutations.

d Sample size used in TL studies after heritability correction and exclusion of patients who were undertaking chemotherapy (see manuscript in results section, TL studies in FBOC).

All cases and controls signed an appropriate informed consent and the proposal was approved by the ethics committee at the Fuenlabrada University Hospital.

We used this set of samples (BRCA1/2 carriers, BRCAX cases and controls) to calculate the percentage of heterozygotes harboring the SNP, to quantify the *OGG1* mRNA levels in peripheral blood, and to perform telomere studies using fresh blood cells, Table 1.

### Lymphoblastoid cell lines

A second set of 23 LCLs was established by Epstein Barr virus transformation of peripheral blood lymphocytes from sixteen healthy women carrying heterozygous mutations in *BRCA1* and seven non-carrier relatives used as controls. Mutational analysis had been previously performed by Sanger sequencing, Supplementary Table S2. None of the women included in the study had personal antecedents of cancer. This LCL panel has been previously described by our group [33]. Cell lines were cultured in RPMI-1640 media (Sigma-Aldrich) supplemented with non-heat-inactivated 20% fetal bovine serum (Sigma-Aldrich), penicillin-streptomycin (Gibco) and Fungizone (Gibco). The cultures were carried out in 25 cm2 flasks (Corning) at 37°C in 5% CO2 atmosphere and cell lines were maintained in exponential growth by daily dilution to 10^6^ cells/ml of full media.

We used this set of samples to measure *OGG1* mRNA expression levels, DNA damage at basal conditions and whenever possible telomere shortening and the percentage of short telomeres gained/lost after 55 passages of cell culture.

### SNP genotyping

The SNP rs2304277, showed the strongest association to cancer risk among all the SNP covering the gene (tagged or imputed) that were included in our previous study [5]. This SNP is located 1.8 kb downstream the 3′UTR (untranslated region) of the gene. Despite we did not find better results for a more plausible causal SNP, we could detect that SNPs in high linkage disequilibrium (LD) with rs2304277, presented similar cancer association direction and p-values [5]. Hence, we considered rs2304277 as a good representative of that gene region, which is detailed in Supplementary Table S5.

DNA was extracted from patient's peripheral blood (FBOC) and from cultured LCLs using MagNAPure LC 2.0 (Roche Diagnostics, Indianapolis, Indiana) following manufacturer's conditions. DNA quantification and quality was assessed by NanoDrop® (ND-1000 V3.7.1).

Flanking region of the rs2304277 was amplified using PCR method with the following primers: *OGG1* “rs2304277-G>A”-F: 5′ GACCTTTCTCGGACCCCATA 3′*OGG1* “rs2304277-G>A”-R: 5′ ACTCCTCCCCAT CCCTACC 3′ and the product was genotyped using Sanger method using ABI3700.

### RNA expression analysis

Using TRIzol Reagent (Ambion®, Life Techonogies) according to manufacturer's instructions, RNA was extracted from peripheral blood cells. Both RNA quantity and quality were assessed by NanoDrop® (ND-1000 V3.7.1).

1 μl of cDNA at a final concentration 10-20 ng/μl was loaded in triplicate, with GoTaq® qPCR MasterMix 1x (Promega); *OGG1* cDNA primers (F/R) and *GAPDH* cDNA primers (F/R) in final concentration of 500nM. All the mentioned reagents were used following manufacture's conditions. Delta Delta Ct method was run in ABI quant studio S7.

cDNA-*OGG1*-F:5′ CTCCACTCCTGCCCTGTG 3′

cDNA-*OGG1*-F:5′ AGAGAAAAGGCATTCGATGG 3′

cDNA-*GAPDH*-F: 5′ CTCCACTCCTGCCCTGTG 3′

cDNA-*GAPDH*-F: 5′ AGAGAAAAGGCATTCGA TGG3′

### Telomere length measurement (TL)

High throughput quantitative fluorescence *in situ* hybridization (HT-QFISH) with automated fluorescence microscopy was performed as previously described [34]. Briefly, Peripheral Blood Mononuclear cells (PBMCs) were separated by Histopaque-1070 (Sigma-Aldrich) gradient centrifugation. Cells were then counted and plated (80 000 – 100 000 cells/well in clear bottomed black-well 96-well plates precoated with 0.001% (poly)L-lysine solution (Sigma-Aldrich, St. Louis, MO) for 30 minutes at 37°C. 4′,6-diamino-2-phenylindole (DAPI) was used for nucleus staining and a fluorescent peptide nucleic acid (PNA) Cy3 probe against telomeric repeats was used for telomere detection. TL values were analyzed using individual telomere spots in a per cell basis (Approximately 90000 telomere spots per sample, which represents around 3500 cells). Fluorescence intensities were then converted into Kb using L5178-R, L5178-S and CCRF-CEM cells as calibration standards, which have stable telomere lengths of 79.7 Kb, 10.3Kb and 7.5 kb, respectively[35]. Samples were analyzed in duplicate, or triplicate in the case of calibration standards. A TL <3Kb was defined as short. The load of short telomeres was estimated as the percentage of short telomeres (number of short telomeres divided by total number of measured telomeres) in each participant.

### DNA damage

LCLs were cultured 4 hours before fixation with 4% paraformaldehyde (Electron Microscopy Sciences, Hatfield, Philadelphia, USA). Two hours before fixation, cells were counted and seeded into a poly-L-lysine-coated (Sigma-Aldrich) μCLEAR bottom 96-well plate (Greiner Bio-One) at a density of 75,000 cells per 100ul full media per well. LCL were then left for 2 hours in order to attach to the surface of the wells, fixed for 15 min at room temperature, permeabilized in 0.5% Triton X-100 in PBS for 20 minutes at 4°C and stained with primary and secondary antibodies and 4′,6-Diamidino-2-phenylindole dihydrochloride (DAPI) to visualize nuclei. To detect γ-H2AX we used mouse monoclonal anti-phospho-histone H2AX antibody (Millipore; #05-636). Alexa Fluor 488 from molecular probes (Invitrogen; #A-11034) was used, and fluorescent images were automatically taken for each well of the 96-well plate using an Opera High-Content Screening System (Perkin Elmer). Pictures were taken under non-saturating conditions using a 40x magnification lens to calculate the γ-H2AX nuclear signal intensity.

### Statistical analysis

Pearson's chi-squared test was used to calculate whether differences in the frequency of the SNP among the FBOC groups were significant, Supplementary Table S1.

Telomere length (Kb) was adjusted to the age, using the best fit line controls (y= −0.067* age (years) +12.785). The difference between the actual and the predicted value was calculated for each sample.

For the comparative analysis we have considered healthy or affected (patients with cancer antecedents) *BRCA1* and *BRCA2* mutation carriers in a single group BRCA1/2. We performed an independent linear regression analysis, using cancer status as a binary variable to test whether it could affect significantly *OGG1* mRNA expression, TL or percentage of short telomeres (Supplementary Table S3).

Kolmogorov-Smirnov test was used to evaluate if the data sets were normally distributed. For the comparative analysis (*OGG1* mRNA expression, Telomere studies and γH2AX nuclear intensity signal), statistically significant differences were assessed by Mann-Whitney U test for not normal distributions (Figures: 1a, 1b, 2a, 2b, 3a, 3b, 4b) and complementary, using lineal regression analysis whenever necessary:

- Regarding the expression studies, lineal regression model including as explanatory variable *OGG1* SNP, was run to test whether this variable could affect *OGG1* mRNA levels in each FBOC group (BRCA1/2 carriers, BRCAX cases and non-carriers controls).
- In relation with the TL studies, a linear regression model was created including as explanatory variables age and the SNP among the different genotypes. Then, if significant differences were found, a separate model was created for each of the genotypes: i) Controls (all) ii) BRCA1/2 carriers harboring the variant iii) BRCA1/2 carriers without the variant. Significant differences among the models were tested with F-test (Figure 2c).

For all the analysis, bilateral p values less than p<0.05 where considered significant.

Statistical calculations were done by SPSS version 18 (SPSSI« Inc, Chicago, Illinois), the R project for statistical computing, GraphPad Prim 5.03 (San Diego, California), and graphics were performed by GraphPad Prim 5.03 (San Diego, California)

## SUPPLEMENTARY FIGURES AND TABLES


