# Supplementary material for: Molecular insights into the OGG1 gene, a cancer risk modifier in BRCA1 and BRCA2 mutations carriers
Source: Oncotarget. 2016 Mar 22;7(18):25815–25. doi: 10.18632/oncotarget.8272 (PMC5041946; doi:10.18632/oncotarget.8272)
Supplement: Supplementary file 1 [file oncotarget-07-25815-s001.pdf]

SUPPLEMENTARY FIGURES AND TABLES

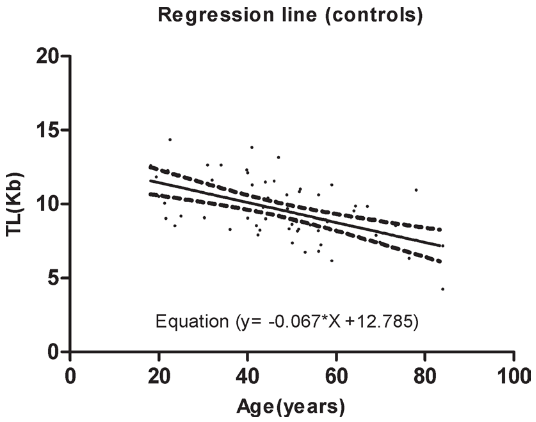

Supplementary Figure S1: TL distribution in peripheral blood leukocytes as a function of age for the control women population (n = 60), measured by HT QFISH. The regression line for control is drawn controls (y= -0.067* age + 12.785).

Supplementary Table S1: Samples distribution and heterozygous frequency of the *OGG1* variant rs2304277

FBOC	Sample size	Heterozygotes frequency (%)
FBOC*	220	
FBOC rs2304277	81	36
Controls	60	
Controls rs2304277	26	43
BRCA1	38	
BRCA1 rs2304277	13	36
BRCA2	48	
BRCA2 rs2304277	16	34
BRCAX	74	
BRCAX rs2304277	26	35

No significant differences were found among the groups. * We have excluded individuals harboring mutations in both *BRCA1* and *BRCA2* genes simultaneously (n=3), none of them harbor the SNP. Hence, the total sample size is 223.

Supplementary Table S2: List of lymphoblastoid cell lines (LCL)

LCL [a]	BRCA1 mutation[b]	Mutation type[c]	Exon	[d]Age	[e]rs2304277	
06S179-L[1]	Wild type	-	-	31	Wt	
09S797-L[2]	Wild type	-	-	27	Wt	
10S889-L[3]	Wild type	-	-	20	Wt	
11S66-L[4]	Wild type	-	-	30	G>A	
11S534-L[5]	Wild type	-	-	50	G>A	
11S954-L	Wild type	-	-	35	Wt	
11S375-L	Wild type	-	-	23	Wt	
05S1303-L[1]	p.Ala1708Glu	Missense	18	59	Wt	*
06S1159-L	c.5123C > A; p.Ala1708Gluf	Missense	18	37	G>A	
10S1202-L	c.5123C > A; p.Ala1708Glu	Missense	18	53	Wt	
10S890-L[3]	c.5123C > A; p.Ala1708Glu	Missense	18	25	Wt	*
11S65-L[6]	c.5117G > A; p.Gly1706Glug	Missense	18	31	G>A	
11S67-L[6]	c.5117G > A; p.Gly1706Glu	Missense	18	34	Wt	
07S1291-L	c.3239T > A; p.Leu1080X	Nonsense/TRC	11	34	G>A	*
09S798-L[2]	c.2410C > T; p.Gln804X	Nonsense/TRC	11	24	Wt	
09S546-L	c.212 + 1G > A; p.?	Splice/TRC	5	42	G>A	*
11S376-L[7]	c.212 + 1G > A; p.?	Splice/TRC	5	39	Wt	*
11S384-L[7]	c.212 + 1G > A; p.?	Splice/TRC	5	75	Wt	*
06S1167-L	c.3331_3334delCAAG; p.Gln1111	Frameshift/TRC	11	33	Wt	
09S491-L	c.815_824dup10; p.Thr276	Frameshift/TRC	11	24	G>A	
10S1177-L[4]	c.68_69delAG; p.Glu23	Frameshift/TRC	2	27	G>A	*
10S44-L	c.4309delT; p.Ser1437	Frameshift/TRC	13	22	Wt	*
11S1004-L[5]	c.981_982delAT; p.Cys328X	Frameshift/TRC	11	25	G>A	

[a] 1–7 LCL from relatives of the same family (sisters or mother & daughter).
[b] Mutation nomenclature based on GenBank reference sequences NM_007294.3 with numbering starting at the A of the first ATG, following the journal guidelines (www.hgvs.org/mutnomen); p.?, unknown protein nomenclature (variant causing skipping of exon 5 of *BRCA1*).
[c] -: Refers to the non-carrier control; TRC: Stands for truncating mutation.
[d] Age of the woman at the time of extraction of the blood sample from which the LCL was established.
[e] G>A indicate the polymorphism (rs2304277).
* For those cells used in the experiment of telomere shortening along 55 passages.

Supplementary Table S3: Lineal regression analysis in BRCA1/2 mutation carriers

Dependent variables	Independent variables	β coeff	p-values	95% C.I ((Lower)-(Upper limit))
OGG1 mRNA	SNP	-0.591	0.027	((-1.113)-(-0.070))
	Cancer	0.148	0.549	((-0.342)-(0.639))
TL(Kb)	SNP	-1.438	0.013	((-2.554)-(-0.323))
	Cancer	-0.115	0.832	((-1.199)-(0.969))
Short telomeres (%)	SNP	-0.030	0.990	((-4.886)-(4.825))
	Cancer	-0.908	0.700	((-5.625-(3.810))

We included as dependent variables *OGG1* mRNA, TL (Kb) and percentage of short telomeres (%), and as independent variables, the SNP and the cancer status. β coefficients quantify how much the 2 independent variables (*OGG1* SNP and cancer status) modify *OGG1* mRNA levels, TL (Kb) and the percentage of short telomeres and also the modification direction. C.I stands for confidence interval.

Supplementary Table S4: Gtex information summary, regarding *OGG1* transcriptional down regulation when rs2304277 is present (5 different tissues)

Gene Symbol	SNP	[a]p-value	Tissue
OGG1	rs2304277	0.57	Cells - EBV-transformed lymphocytes
OGG1	rs2304277	0.22	Uterus
OGG1	rs2304277	0.16	Vagina
OGG1	rs2304277	0.45	Whole Blood
OGG1	rs2304277	0.023	Ovary

[a]Nominal eQTL p-values were generated for each SNP-gene pair using a two-tailed t test, testing the alternative hypothesis that the beta (slope of the linear regression model) deviates from the null hypothesis of $\beta=0$.

Supplementary Table S5: Table with information regarding the SNPs within the block of Linkage disequilibrium (LD) >0.8 with the SNP rs2304277

SNP in LD>0.8 with rs2304277			
**SNPs**	**Gene**	**Location***	**r^2(LD)**
rs3219008	*OGG1*	intronic	0.86
rs2075747	*OGG1*	intronic	0.93
rs2072668	*OGG1*	intronic	0.85
rs1052133	*OGG1*	Missense/ 3-UTR	0.83
rs4021704	*OGG1/ CAMK1*	intronic	0.98
rs2304277	*OGG1/ CAMK1*	Downstream 3-UTR(*OGG1*)	*1.00*
rs7609858	*OGG1/ CAMK1*	intronic	0.99
rs6763347	*OGG1/ CAMK1*	intronic	1.00
rs73021455	*OGG1/ CAMK1*	intronic	0.90
rs66482970	-	-	0.89
rs57081507	-	-	0.89
rs67055061	-	-	0.90
rs14204	TADA3	3'-UTR	0.83
rs6809452	*TADA3*	intronic	0.88
rs7610826	*TADA3*	intronic	0.87
rs7618535	*TADA3*	intronic	0.87
rs7618636	*TADA3*	intronic	0.88
rs7621556	*TADA3*	intronic	0.88

*In some cases the SNP can cover more than 1 location depending on the gene isoform. (due to limited space only one gene location is available at the table).
